# Special Issue “Structural Dynamics of Macromolecules”

**DOI:** 10.3390/ijms27020961

**Published:** 2026-01-18

**Authors:** Ki Hyun Nam

**Affiliations:** College of General Education, Kookmin University, Seoul 02707, Republic of Korea; structure@kookmin.ac.kr

Understanding the molecular function of macromolecules is fundamental to understanding their biological roles [1,2]. In particular, macromolecular structures provide intuitive insights into their functions, including how biomolecules interact with substrates, ligands, and other biomolecules [3,4]. Various biotechnical techniques, such as X-ray crystallography, nuclear magnetic resonance (NMR), cryo-electron microscopy (cryo-EM), and micro-electron diffraction (microED), have been widely used to analyze high-resolution molecular structures [5,6,7] and provide insights into molecular flexibility and conformational states, while also being potentially useful for understanding the mechanisms of action of biological macromolecules [8,9,10]. In addition, time-resolved serial crystallography using X-ray free-electron lasers (XFELs) or synchrotron radiation, as well as time-resolved cryo-EM, provide structural insights into the dynamics of biomolecules during reaction processes [11,12,13,14]. Furthermore, molecular dynamics (MD) simulations can be used to obtain information on the motions and dynamics of macromolecules that are experimentally challenging to characterize [15]. These structural data not only offer valuable information for accurately understanding biological functions but also provide insights into protein engineering in the fields of drug discovery and industrial applications [16,17]. This Special Issue includes structural analyses of biological macromolecules, related technical and computational advancements, enzymatic mechanisms and industrial applications, and regulation and evolution, providing an important foundation for understanding macromolecular functions.

Makarov and Kharlamova demonstrate that pulse length is the dominant parameter governing the scattering of attosecond ultrashort pulses from DNA and RNA trinucleotides, leading to diffraction patterns that differ substantially from those predicted by conventional scattering theory. These findings suggest that time-resolved X-ray diffraction using attosecond pulses requires pulse-duration-dependent scattering models to correctly interpret and decode DNA and RNA structures.

Bu et al. reported the crystal structure of L-methionine γ-lyase (MGL) in *Fusobacterium nucleatum*, a lesion-associated obligate anaerobic pathogen implicated in destructive periodontal disease. Their structural analysis provides mechanistic insights into the molecular function of MGL and offers a structural basis for the rational design of potential inhibitors targeting this enzyme.

Bai et al. provide a biochemical and structural characterization of butanol dehydrogenase (BDH) from *Fusobacterium nucleatum*, highlighting its potential relevance for butanol biosynthesis. Their study identified substrate specificity, cofactor dependence, and key residues involved in substrate and cofactor binding, providing mechanistic insights into the molecular function of BDH and a structural basis for protein engineering toward industrial applications.

Yang et al. provided a structural characterization of 3-hydroxybutyryl-CoA dehydrogenase (A2HBD) from *Faecalibacterium prausnitzii* A2-165, a key enzyme involved in the butyrate production pathway associated with atopic dermatitis. They elucidated the cofactor and substrate binding modes, substrate-induced conformational changes, and key residues involved in catalysis, providing mechanistic insights into enzyme function and a structural basis for understanding butyrate biosynthesis.

Wu et al. reported on the novel homodimeric crystal structure of the N-terminal zinc finger motifs (Zif1–2) of the transcription factor GLI1, which is distinct from the DNA-bound conformation. Structural and computational analyses suggest that this dimeric arrangement may represent the native apo form of GLI1, providing new insights into its DNA-binding regulation.

Kim et al. demonstrated the effect of symmetry parameter selection in cryo-EM single-particle analysis by reconstructing the E2 inner core of the pyruvate dehydrogenase complex under multiple symmetry assumptions. The results show that near-atomic structures can appear identical despite different processing parameters, highlighting that improper parameter choices may obscure biologically meaningful dynamic information and require careful interpretation.

Hinostroza et al. utilized all-atom molecular dynamics simulations to investigate how infertility-associated mutations in sperm-specific phospholipase Cζ disrupt its function during fertilization. The results show that H233L and H398P mutations impair PIP2 and Ca^2+^ binding through altered interactions and substrate misalignment, whereas R553P mainly affects structural stability and dynamics, providing a molecular explanation for defective egg activation and male infertility.

Wang et al. systematically classified of ribose 2′-O-methyltransferases (2′-O-MTases) into 11 families and revealed their widespread distribution across the Tree of Life, with several families tracing back to the Last Universal Common Ancestor. Evolutionary and structural analyses showed that most 2′-O-MTases are conserved under purifying selection while undergoing lineage-specific expansion and functional diversification, particularly exemplified by the FtsJ family specializing in distinct RNA substrates.

A wide range of macromolecule-related studies are included in this Special Issue, addressing structural dynamics and evolutionary perspectives in addition to macromolecular structures. The contributions extend from fundamental science to applied research, and together they provide valuable insights that will facilitate a deeper understanding of the related fields.
